# A Reversible Shift of Driver Dependence from EGFR to Notch1 in Non-Small Cell Lung Cancer as a Cause of Resistance to Tyrosine Kinase Inhibitors

**DOI:** 10.3390/cancers13092022

**Published:** 2021-04-22

**Authors:** Francesca Iommelli, Viviana De Rosa, Cristina Terlizzi, Rosa Fonti, Rosa Camerlingo, Maria Patrizia Stoppelli, C. Allison Stewart, Lauren Averett Byers, David Piwnica-Worms, Silvana Del Vecchio

**Affiliations:** 1 Institute of Biostructures and Bioimaging, National Research Council, 80145 Naples, Italy; francesca.iommelli@ibb.cnr.it (F.I.); viviana.derosa@ibb.cnr.it (V.D.R.); rosa.fonti@ibb.cnr.it (R.F.); 2 Department of Advanced Biomedical Sciences, University “Federico II”, 80131 Naples, Italy; cr.terlizzi88@gmail.com; 3 Department of Cell Biology and Biotherapy, Istituto Nazionale Tumori-IRCCS-Fondazione G. Pascale, 80131 Naples, Italy; r.camerlingo@istitutotumori.na.it; 4 Institute of Genetics and Biophysics, “Adriano Buzzati Traverso” National Research Council, 80131 Naples, Italy; mpatrizia.stoppelli@igb.cnr.it; 5 Department of Thoracic Head and Neck Medical Oncology, The University of Texas MD Anderson Cancer Center, Houston, TX 77030, USA; astewart@mdanderson.org (C.A.S.); lbyers@mdanderson.org (L.A.B.); 6 Department of Cancer Systems Imaging, The University of Texas MD Anderson Cancer Center, Houston, TX 77030, USA; dpiwnica-worms@mdanderson.org

**Keywords:** Notch1, EGFR, EMT, tumor spheres, NSCLC

## Abstract

**Simple Summary:**

Notch1 and EGFR are two surface receptors activating different cellular processes in cancer cells. EGFR, harboring activating mutations, drives unlimited cell proliferation in non-small cell lung cancer (NSCLC), and treatment of tumors with tyrosine kinase inhibitors (TKIs) results in growth arrest and cell death. On the other hand, Notch1 plays a key role in the loss of epithelial characteristics and the concomitant acquisition of mesenchymal traits and invasive potential of tumor cells. Interestingly, high levels of Notch1 are associated with the resistance to EGFR TKIs. Here, we evaluated the mechanisms by which Notch1 causes resistance of NSCLC to EGFR TKIs, and provided evidence that high levels of activated Notch1 induce a decrease of EGFR, by modulating the activity of the promoter of the *EGFR* gene. Therefore, blocking the Notch1 pathway in tumors treated with EGFR inhibitors would prevent EGFR downregulation, maintaining drug sensitivity.

**Abstract:**

Notch1 plays a key role in epithelial-mesenchymal transition (EMT) and in the maintenance of cancer stem cells. In the present study we tested whether high levels of activated Notch1 in oncogene-driven NSCLC can induce a reversible shift of driver dependence from EGFR to Notch1, and thus causing resistance to EGFR inhibitors. Adherent cells (parental) and tumor spheres (TS) from NSCLC H1975 cells and patient-derived CD133-positive cells were tested for EGFR and Notch1 signaling cascade. The Notch1-dependent modulation of EGFR, NCID, Hes1, p53, and Sp1 were then analyzed in parental cells by binding assays with a Notch1 agonist, DLL4. TS were more resistant than parental cells to EGFR inhibitors. A strong upregulation of Notch1 and a concomitant downregulation of EGFR were observed in TS compared to parental cells. Parental cell exposure to DLL4 showed a dose-dependent decrease of EGFR and a simultaneous increase of NCID, Hes1, p53, and Sp1, along with the dislocation of Sp1 from the *EGFR* promoter. Furthermore, an enhanced interaction between p53 and Sp1 was observed in TS. In NSCLC cells, high levels of active Notch1 can promote a reversible shift of driver dependence from EGFR to Notch1, leading to resistance to EGFR inhibitors.

## 1. Introduction

Multiple molecular mechanisms may cause resistance of cancer cells to targeted agents, such as tyrosine kinase inhibitors (TKIs), and the development of novel therapeutic strategies that are able to overcome or prevent resistance is one of the main clinical challenges in oncology [1,2]. In response to a prolonged inhibition of oncogene drivers, cancer cells may adopt compensatory pathways that maintain the mitogenic cascade as persistently activated [3,4]. Alternatively, the target may acquire genetic alterations that prevent drug binding, thus leading to treatment failure. An additional determinant of resistance to targeted agents is the heterogeneity of tumor cells [5,6] due to the presence of a sub-population of cancer stem cells (CSCs) and to the occurrence of epithelial-mesenchymal transition (EMT) [7,8]. CSCs are characterized by an unlimited proliferative potential, an extended self-renewal and differentiation ability, and a high degree of cell plasticity, thus promoting cancer initiation, maintenance, and progression [9]. Another common property of CSCs is their highly inherited resistance to chemotherapeutic drugs, targeted agents, and radiotherapy, due to not fully elucidated molecular mechanisms, including an altered apoptotic program, a quiescent state, enhanced drug efflux, or hypoxic microenvironmental conditions. Resistant CSCs, with their cancer initiation ability, may serve as precursors of new tumor masses, leading to clinical relapse, even after complete tumor response to treatment [7,10].

EMT is a reversible cellular process leading to the loss of epithelial characteristics and the concomitant acquisition of mesenchymal traits [8,11], high cell mobility, and an invasive phenotype [8,12]. Furthermore, several studies have shown an association between EMT and the acquisition of stem-like properties, such as an elevated tumor-initiating potential, expression of distinct cell surface markers (including CD44 and CD133), and inherent drug resistance [13]. Although it is not clear how the EMT process is linked to the CSC state, the two biological programs are thought to be closely associated, since they share common signaling pathways. In particular, TGFβ, WNT, and Notch signaling cascades have been reported to induce the activation of a pool of transcription factors that in turn mediate the execution of the EMT program [12].

In patients with non-small cell lung cancer (NSCLC), Notch1 is reported to have a dominant role in the activation of the EMT program and an association with drug resistance [14]. The four isoforms of Notch receptor (Notch1–Notch4) are activated upon binding to several ligands, such as JAG1, JAG2, DLL1, and DLL4, expressed on the surface of neighboring cells [15]. The interaction of the receptor with its cognate ligand induces a conformational change, followed by cleavage of the Notch intracellular domain (NICD) by the presenilin-γ-secretase complex and its subsequent translocation to the nucleus. Then, NICD converts the CBF1-Su(H)-LAG1 (CSL) repressor complex into a transcriptional activator complex, driving the transcription of several target genes, including *cyclin D1, Myc, Bcl-2, Hes1,* and *Hey1,* involved in cancer cell cycle progression, as well as the inhibition of apoptosis and metastasis formation. High levels of Notch1 were found in human NSCLC and were associated with drug resistance and poor prognosis [14,16]. In particular, the upregulation of Notch1 was observed in oncogene-driven NSCLC resistance to EGFR inhibitors in association with high levels of SNAIL, SLUG, and Twist [17,18,19].

The aim of the present study was to test whether high levels of activated Notch1 can induce a shift of driver dependence, from EGFR to Notch1, in oncogene-driven NSCLC, and thus causing resistance to EGFR inhibitors. Furthermore, the molecular mechanisms underlying such a shift were investigated, with the purpose of identifying the transcriptional factors responsible for driver exchange between EGFR and Notch1.

## 2. Results

### 2.1. Tumor Sphere Formation and Expression of Stemness Markers

**Figure 1:**
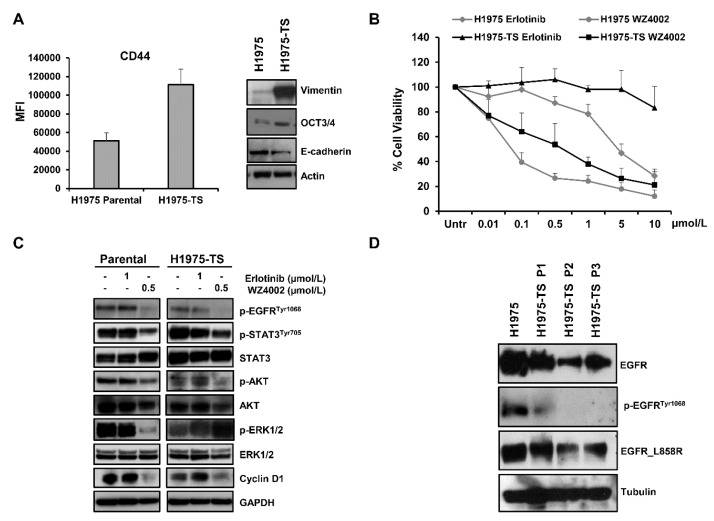
Levels of stemness and EMT markers in H1975 NSCLC cells grown in adhesion or as tumor spheres, and response to EGFR inhibitors. (**A**) Levels of CD44, vimentin, OCT3/4, and E-cadherin in H1975 parental cells and H1975 tumor spheres (H1975-TS). (**B**) Cell toxicity assay in parental cells and H1975-TS exposed to increasing concentrations of erlotinib and WZ4002 for 72 h. (**C**) Levels of EGFR downstream signaling mediators and effectors in response to treatment with EGFR inhibitors (0.5 or 1 µmol/L) for 48 h. (**D**) Levels of total EGFR, p-EGFR^Tyr1068^, and EGFR_L858R in parental cells and tumor spheres at different serial passages. At least three independent experiments were performed. Actin, GAPDH, or tubulin were used to ensure equal loading.

NSCLC H1975 cells were cultured in serum-free, non-adherent conditions and tumor spheres (H1975-TS) were obtained after approximately 2 weeks of cell growth. The progressive enrichment of stem-like tumor cells was achieved after at least three passages of spheroids. Increased levels of CD44, vimentin, and OCT3/4, along with a decreased expression of E-cadherin were found in tumor spheres compared to parental cells (Figure 1A), indicating the occurrence of an epithelial mesenchymal transition (EMT) and/or the acquisition of a stem-like phenotype. Since parental H1975 cells expressed EGFR receptor with activating point mutation L858R and secondary T790M mutation, we tested whether tumor spheres maintained the same EGFR mutations. An RT–PCR array for human EGFR pathway did not show any significant difference in the *EGFR* gene, EGFR mutational status, or eight additional key genes in the EGFR pathway between parental cells and H1975-TS (Supplementary Figure S1).

### 2.2. Effects of EGFR Inhibitors in Parental Cells and Tumor Spheres

Cell toxicity was assessed by MTS assays in parental cells and H1975-TS exposed to increasing concentrations of erlotinib and WZ4002 for 72 h (Figure 1B). As expected, H1975 parental cells showed resistance to erlotinib and a high sensitivity to WZ4002. In contrast, the H1975-TS exhibited an increased erlotinib resistance and were less sensitive than parental cells to WZ4002. In agreement with these findings, levels of the phosphorylated form of STAT3^Tyr705^, AKT, and ERK ½, as well as cyclin D1 were unchanged in both parental cells and tumor spheres in response to erlotinib, whereas treatment with WZ4002 caused a more pronounced decrease of these signaling mediators in parental cells compared to tumor spheres, except for p-AKT (Figure 1C). In order to test whether changes in the levels of total EGFR occurred during tumor sphere formation and serial passages, whole cell lysates of parental cells and tumor spheres at passage 1–3 were analyzed by western blot. A progressive decrease of total EGFR levels was observed in H1975-TS during serial passages compared to parental cells (Figure 1D), along with its phosphorylated form. Despite the decreased levels of EGFR in H1975-TS, the receptor maintained the activating mutations in its kinase domain.

### 2.3. Overexpression and Activation of Notch1 in Tumor Spheres

**Figure 2:**
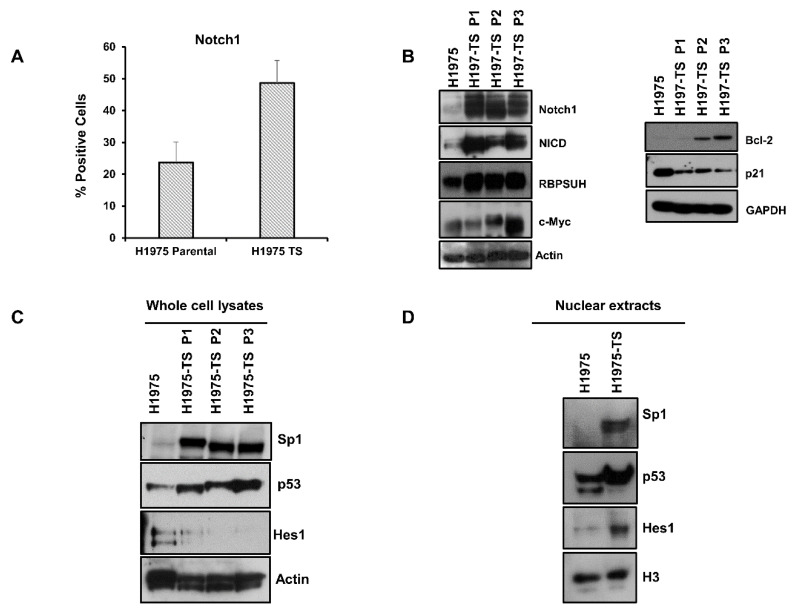
Notch1 expression and activation in H1975 parental cells and tumor spheres at different serial passages. (**A**) FACS analysis of Notch1 expression on the plasma membrane of H1975 cells and H1975-TS. (**B**) Levels of Notch1, Notch intracellular domain (NICD), RBPSUH, c-Myc, Bcl-2, and p21 in whole cell lysates of H1975 and H1975-TS at different serial passages. (**C**,**D**) Levels of Sp1, p53, and Hes1 transcription factors in whole cell lysates (**C**) and nuclear extracts (**D**) of H1975 and H1975-TS. At least three independent experiments were performed. Actin, GAPDH, or histone H3 were used to ensure equal loading.

Since Notch1 plays a key role in EMT and in the maintenance of CSCs, we tested whether levels and activation of Notch1 were enhanced in H1975-TS compared to parental cells. Flow cytometry showed increased levels of Notch1 on the plasma membrane of cells grown in tumor spheres (Figure 2A). A strong upregulation of Notch1 and Notch intracellular domain (NICD), i.e., the cleaved and active form of Notch1, was found in whole cell lysates of tumor spheres at serial passages, along with increased levels of Notch1 effectors, such as RBPSUH and c-Myc (Figure 2B). Furthermore, the enhanced expression and activation of Notch1 was associated with a passage-dependent increase of Bcl-2 levels and downregulation of p21, thus promoting survival and cell cycle progression of tumor spheres (Figure 2B).

To identify the factors involved in the coordinated and differential regulation of Notch1 and EGFR, we tested the levels of key transcription factors having binding sites in the promoter of both *Notch1* and *EGFR* genes. High levels of Sp1 and p53 were found in both whole cell lysates and nuclear extracts of H1975-TS, whereas they were barely detected in samples of parental cells (Figure 2C,D). Furthermore, increased levels of Hes1 were found in nuclear extracts of tumor spheres compared to parental cells, whereas whole cell lysates showed the highest levels of Hes1 in parental cells, indicating a nuclear translocation of this signaling mediator during the acquisition of the stem-like phenotype.

### 2.4. Notch1-Dependent Downregulation of EGFR in Parental Cells

**Figure 3:**
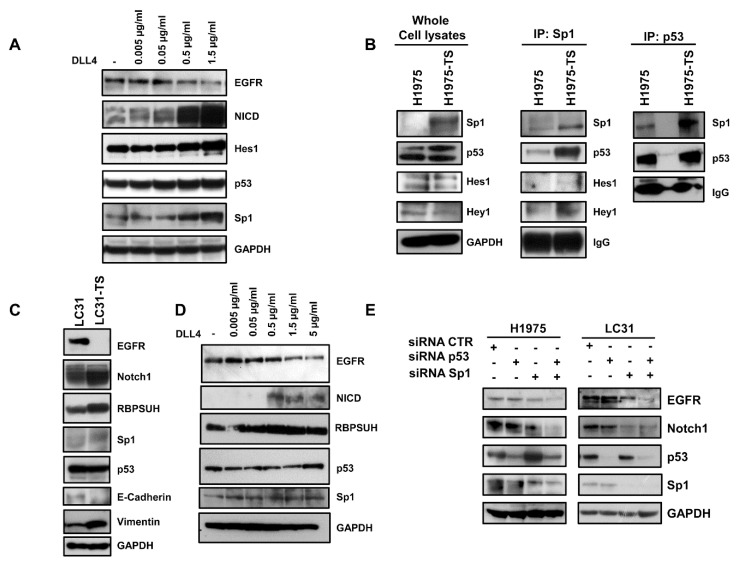
Downregulation of EGFR in response to DLL4 binding in H1975 and LC31 parental cells and modulation of Sp1 and p53 transcription factors. (**A**) Dose-dependent decrease of EGFR levels in response to the DLL4 stimulation for 72 h of H1975 parental cells, and the concomitant activation of Notch1 signalling cascade and levels of p53 and Sp1. (**B**) Direct or indirect interaction between Sp1 and p53 in whole cell lysates from H1975 and H1975-TS assessed by immunoprecipitation with Sp1 or p53 antibodies. (**C**) Levels of EGFR, Notch1, RBPSUH, Sp1, p53, and EMT markers in parental LC31 and LC31 tumor spheres (TS). (**D**) Dose-dependent decrease of EGFR levels in response to DLL4 stimulation for 72 h of parental LC31 cells, concomitant activation of Notch1 signalling cascade and levels of p53 and Sp1. (**E**) H1975 and LC31 parental cells were transfected with siRNA scrambled (CTR) Sp1- and p53-targeted siRNAs, either alone or in combination for 72 h, and levels of EGFR and Notch1 were determined by western blot analysis. At least three independent experiments were performed. GAPDH was used to ensure equal loading.

To test whether the downregulation of EGFR in tumor spheres was dependent on the activation of Notch1, we performed binding assays of parental cells (H1975 and HCC827) to plates coated with an increasing concentration of the DLL4 Notch agonist. A dose-dependent decrease of EGFR levels was observed in response to Notch1 activation, as shown by the progressive increase of NICD levels (Figure 3A and Supplementary Figure S2). Furthermore, a Notch-dependent upregulation of Hes1, p53, and Sp1 was also observed in parental cells, indicating a potential role of these transcription factors in EGFR downregulation in tumor spheres. Moreover, binding of H1975 cells to DLL4 showed a reduction of sensitivity to WZ4002, as assessed by MTS (Supplementary Figure S3).

Next, we tested the physical interaction between the transcription factors modulated by Notch1 activation. Immunoprecipitation assays showed an enhanced interaction between Sp1 and p53 in whole cell lysates of tumor spheres compared to samples of parental cells (Figure 3B). A slight increase of the interaction between Sp1 and Hey1 was also observed. The enhanced interaction between Sp1 and p53 in tumor spheres was confirmed by the immunoprecipitation of whole cell lysates with anti-p53 antibody.

To test whether the activation of Notch1 caused downregulation of EGFR, even in CSCs derived from human specimens of NSCLC, LC31 cells growing in adhesion or as tumor spheres (LC31-TS) were preliminarily characterized and then subjected to binding assays to DLL4-coated plates. A strong reduction of EGFR levels was observed in LC31-TS compared to parental cells (Figure 3C). Furthermore, LC31-TS showed an upregulation of Notch1, RBPSUH, Sp1, and vimentin, along with a reduction of E-cadherin levels (Figure 3C). When parental LC31 cells were incubated for 72 h in DLL4-coated plates, a dose-dependent decrease of EGFR levels was observed in response to Notch1 activation, along with increased levels of NICD and RBPSUH. Furthermore, a mild increase of p53 and Sp1 was also observed in LC31 upon binding to DLL4 (Figure 3D).

### 2.5. RNA Interference and ChIP

**Figure 4:**
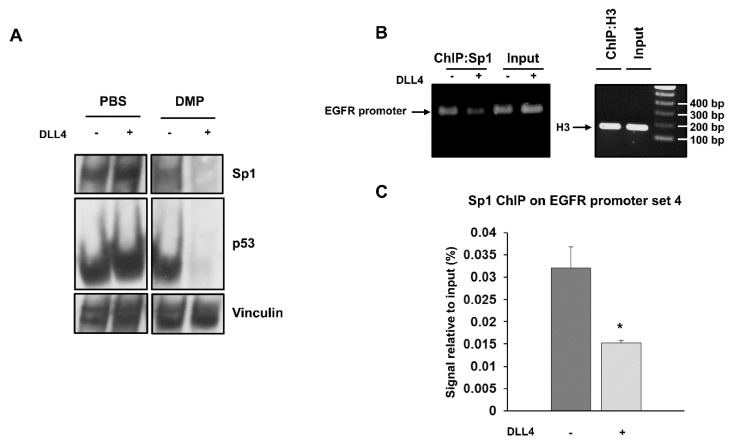
Cross-linking of Sp1 and p53 after DLL4 Notch1 activation and dislocation of Sp1 from *EGFR* promoter assessed by ChIP. (**A**) Cross-linking assay with DMP agent was performed in unstimulated and DLL4-stimulated H1975 cells, and whole cell lysates were analyzed by western blot to detect the free forms of Sp1 and p53. Vinculin was used to ensure equal loading. (**B**) ChIP assay was performed by chromatin immunoprecipitation with an anti-Sp1 antibody in unstimulated and DLL4-stimulated H1975 cells. Immunoprecipitated DNA fragments were then amplified using *EGFR* promoter specific primers set 4. Histone H3 was used as positive control. Levels of amplified products were analyzed on a 2% agarose gel. (**C**) Quantitative analysis of ChIP assay was performed by RT-PCR. Data were expressed as % of input in unstimulated and DLL4-stimulated H1975 cells. At least three independent experiments were performed and data are expressed as mean ± SE. The symbol *, *p* < 0.05.

To test whether p53 and Sp1 levels were critical for the transcription of both *EGFR* and *Notch1*, H1975 and LC31 parental cells were transfected with Sp1- and p53-targeted siRNAs, either alone or in combination, and levels of EGFR and Notch1 were determined by western blot analysis (Figure 3E). Silencing of p53 did not cause any significant changes in either EGFR or Notch1 levels in H1975 and LC31 cells. Downregulation of Sp1 reduced the levels of EGFR and Notch1 in both cell lines. Interestingly, the concomitant silencing of Sp1 and p53 caused an enhanced reduction of EGFR and Notch1 levels compared to those obtained after transfection with Sp1- and p53-targeted siRNA alone. These findings indicate that Sp1 is critical for the expression of EGFR and Notch1, and that p53 levels may potentiate Sp1 transcriptional activity. Furthermore, to confirm the involvement of p53 and Sp1 in a large complex formation in response to Notch1 activation, cross-linking experiments were performed using a membrane-permeable DMP crosslinking agent in unstimulated and DLL4-stimulated parental H1975 cells. The exposure to Notch agonist caused a strong reduction of the free forms of p53 and Sp1 in whole cell lysates from cells incubated with DMP (Figure 4A). Therefore, we reasoned that Notch1 activation in parental cells, by enhancing the expression of p53 and Sp1, may promote their direct or indirect interaction and the resulting complexes may drive the transcription of Notch1 more efficiently than EGFR. This would result in a dislocation of Sp1 and p53 from the *EGFR* promoter. To test the occurrence of Sp1 dislocation, chromatin immunoprecipitation assays (ChIP) were performed in unstimulated and DLL4-stimulated parental H1975 cells, using *EGFR* promoter specific primers recognizing DNA sequences with Sp1 (sets 1 and 4) and p53 (sets 2–4) binding sites [20]. Figure 4B shows that DLL4 stimulation caused a reduction of Sp1 binding to the *EGFR* promoter, as assessed by the reduced amplification product of the Sp1 immunoprecipitated DNA fragment using primer set 4. No differences were found using primer sets 1–3, (Supplementary Figure S4). Real-time PCR showed a two-fold significant decrease (*p* < 0.05) in the amplification of ChIP samples from DLL4-stimulated cells compared to unstimulated cells using primer set 4 (Figure 4C).

## 3. Discussion

The present study shows that high levels of activated Notch1 in oncogene-driven NSCLC cells induce a reversible shift of driver dependence, from EGFR to Notch1, which is associated with the activation of the EMT program and downregulation of EGFR. The reduced or absent expression of the target would cause a reversible resistance to all EGFR inhibitors and antagonists. The mechanisms underlying the inverse modulation of EGFR and Notch1 involve Sp1 and p53, since activation of Notch1 by binding with the agonist DLL4 enhances the expression of Sp1 and p53, promoting their interaction, as assessed by immunoprecipitation assays in H1975 cells. The resulting complexes may cause a dislocation of Sp1 and p53 from the *EGFR* promoter, as shown by ChIP assay, accounting for the downregulation of this receptor. These findings may have important clinical implications, since the treatment of EGFR-driven NSCLC with EGFR inhibitors may cause the upregulation and enhanced activation of Notch1 and Notch3 [18,21], thus promoting the reversible shift of driver dependence from EGFR to Notch.

Previous studies reported that NSCLC cells and tumors with an EMT signature of 76 genes associated with a mesenchymal phenotype were more resistant to EGFR and PI3K/AKT inhibitors [22]. Furthermore, the EMT score of this signature was predictive of erlotinib sensitivity in both EGFR-mutant and EGFR wild-type NSCLC. Our findings are in agreement with these observations, since the expression of EMT markers in tumor spheres was associated with a higher resistance to erlotinib and irreversible EGFR inhibitors. In our study, the activation of the EMT program in both EGFR-mutant and EGFR wild-type NSCLC cells caused a downregulation of EGFR, with the consequent lack of response to targeted inhibitors. Moreover, genetic and histological analysis of tumor biopsies from NSCLC patients with drug resistant tumors showed that all tumors maintained their original activating EGFR mutations and a fraction of them showed a pronounced epithelial-to-mesenchymal transition [23]. Furthermore, in patients with acquired T790M-mediated resistance, the suspension of TKIs treatment was followed by the genetic loss of T790M secondary mutation, and tumors re-acquired the sensitivity to TKIs [23]. Our findings may explain the genetic loss of T790M mutation after drug suspension. In fact, the activation of the EMT program and the acquisition of a mesenchymal phenotype can occur from the beginning of treatment, when tumor cells are still sensitive and do not harbor T790M mutation. Drug suspension may cause the mesenchymal-to-epithelial transition of this stem-like subpopulation, with the re-expression of EGFR bearing activating mutations without T790M secondary mutation.

Notch1 plays a key role in controlling differentiation and proliferation during embryonic development, normal tissue homeostasis, and oncogenic transformation [15]. It is reported to be one of the main drivers of the EMT program in several different types of carcinoma, such as breast, lung, and pancreatic carcinomas [8,15]. The activation of the Notch1 pathway leads to modulation of a panel of transcriptional factors, including Snail, Slug, Twist1, Zeb1, and Zeb2, which constitute the core of the EMT transcriptional machinery. How the different EMT transcriptional factors are regulated, exert their specific action, and orchestrate physiological and pathological processes is not completely understood. Beyond the role of Notch1 to activate core EMT transcription factors, our study highlighted its ability to enroll Sp1 and p53 in large molecular complexes. Once sequestered in these complexes, Sp1 and p53 may not be able to activate the *EGFR* promoter. It is likely that other signaling pathways that activate the EMT program, such TGFβ and WNT, may cause a similar dislocation of transcriptional factors from the promoter of certain oncogenes or genes characterizing the epithelial phenotype.

## 4. Materials and Methods

### 4.1. Cell Culture

NSCLC cell lines H1975 and HCCC827 were obtained from, and authenticated by, American Type Culture Collection (Rockville, MD, USA). Cells were grown in RPMI medium, containing 10% fetal bovine serum, 100 IU/mL penicillin, and 50 µg/mL streptomycin in a humidified incubator in 5% CO_2_ at 37 °C. H1975 cells bear an activating point mutation in exon 21 (L858R) and also harbour the T790M mutation in the kinase domain of EGFR, which is known to confer resistance to erlotinib [24]. In addition, a gain of function of mutant p53 (p53-R273H) has also been reported for these cells [20,25]. HCC827 cells bear an activating drug sensitive *EGFR* mutation (deletion in exon 19, delE746_A750). A human CD133-positive immortalized NSCLC cell line (LC31) was kindly provided by Dr R. Camerlingo, who had previously described cell line isolation from primary NSCLC specimens [26,27]. LC31 cells, subjected to RT–PCR (qBiomarker Somatic Mutation PCR array for human *EGFR* pathway, Qiagen), showed wild type *EGFR* and mutant *RAS* (Supplementary Figure S5). They were grown in IMDM containing 10% FBS 100 IU/mL penicillin, and 50 µg/mL streptomycin in a humidified incubator in 5% CO_2_ at 37 °C.

### 4.2. Tumor Spheres Formation

NSCLC cells were cultured in serum-free, non-adherent conditions, in order to enrich the CSC population and induce the formation of tumor spheres. Briefly, cells were plated in ultralow-attachment multi-well plates at a density of 50,000–60,000 viable cells/well in serum-free medium containing 10 ng/mL of epidermal growth factor (EGF) (BD Biosciences, Franklin Lakes, NJ, USA), 10 ng/mL basic fibroblast growth factor (FGF) (BD Biosciences), and B27 supplement (Gibco). Fresh medium was added every 2–3 days. Cells were allowed to grow for approximately 15–20 days, and tumor sphere formation was observed by inverted light microscopy. Then, first generation tumor spheres were collected by gentle centrifugation and enzymatically dissociated by addition of Accutase solution (Millipore, Burlington, MA, USA). After dissociation, cells were plated to allow formation of second generation tumor spheres and at least three passages were performed to enrich the final CSC population.

### 4.3. Cell Treatment and Toxicity Assay

H1975 and LC31 cells, grown in adhesion or as tumor spheres, were seeded in 96-well flat-bottomed plates at a density of 5000 per well and incubated for 72 h with increasing concentrations (range 0.01–10 µmol/L) of erlotinib or WZ4002, a selective inhibitor of T790M mutant EGFR, in a humidified incubator with 5% CO_2_ at 37 °C. In parallel experiments, H1975 cells were preliminarily exposed to DLL4 and then treated with WZ4002. Drug-induced toxicity was assessed by the MTS assay (Promega, Madison, WI, USA), as previously described [4]. After the addition of MTS, the number of viable cells was determined spectrophotometrically and expressed as the percentage of viable cells, considering the untreated control cells as 100%. At least three independent experiments were performed in triplicates, and data were pooled.

### 4.4. FACS Analysis

Levels of CD44 and Notch1 on the plasma membrane of NSCLC cells were determined by fluorescence-activated cell sorting (BD FACSAria II, Franklin Lakes, NJ, USA). Briefly, cells were incubated with PE-conjugated mouse antibodies against Notch1 (Biolegend, San Diego, CA, USA) and CD44 (eBioscience, San Diego, CA, USA) for 1 h at 4 °C in the dark and then washed with cold PBS. Then cells were subjected to FACS analysis using BD FACSDiva 8.0 software (8.0 version, BD Biosciences, Franklin Lakes, NJ, USA). At least three independent experiments were performed for each cell surface marker.

### 4.5. Western Blot and Nuclear Extraction

Whole cell lysates were prepared from cells grown in adhesion or as tumor spheres as previously described [4,24]. Briefly, cells were treated with erlotinib or WZ4002 (0.5 or 1 µmol/L) for 48 h at 37 °C. Untreated and treated cells were lysed on ice in RIPA buffer, with protease and phosphatase inhibitors (Sigma-Aldrich, St. Louis, MO, USA). The suspension was then homogenized and centrifuged at 13,000× *g* for 30 min at 4 °C.

For nuclear protein extraction [28], cells were washed twice in ice-cold PBS and the pellets were suspended in 1 mL of buffer A (10 mmol/L HEPES pH 7.9, 1.5 mmol/L MgCl_2_, 10 mmol/L KCl, 0.01% Triton X-100, 0.5 mmol/L DTT, and 0.5 mmol/L PMSF). After 20 min on ice, samples were vortexed and nuclei were recovered by centrifugation for 5 min at 5000 rpm at 4 °C, followed by resuspension in 25 μL of buffer B (10 mmol/L HEPES pH 7.9, 1.5 mmol/L MgCl_2_, 400 mmol/L KCl, 0.2 mmol/L EDTA, 25% glycerol, 0.5 mmol/L DTT, protease inhibitors, and 0.5 mmol/L PMSF). After an additional 20 min on ice, samples were subjected to two cycles of freeze–thaw in liquid nitrogen and then maintained at 4 °C for 5 min. Nuclear extracts were centrifuged for 10 min at 13,000 rpm at 4 °C, and the supernatant was recovered. Western blotting of proteins from both whole cell lysates and nuclear extracts was carried out using a standard procedure. PVDF membranes were probed by using mouse monoclonal antibodies recognizing p-EGFR^Tyr1068^, p42/44 MAP kinase, STAT3 (Cell Signaling, Danvers, MA, USA), vimentin (Abcam, Cambridge, UK), e-cadherin, Bcl-2, p53 (DO-1) (Santa Cruz Biotechnologies, Dallas, TX, USA), OCT-3/4 (BD Transduction Laboratories), α-tubulin, actin (Sigma), rabbit monoclonal antibody GAPDH, EGFR (L858R mutant specific), p-STAT3^Tyr705^, Hes1, Notch1 (D1E11), cleaved Notch1 (Val1744), Sp1 (D4C3), p21 Waf1/Cip1, and RBPSUH (D10A4) (Cell Signaling), and rabbit polyclonal antibodies specific for EGFR, p-AKT^Ser473^ (Santa Cruz Biotechnology, Dallas, TX, USA), AKT, p-p42/44 MAP kinase (Thr202/Tyr204), cyclin D1, c-Myc (Cell Signaling), Hey1 (ThermoFisher Scientific, Waltham, MA, USA), and histone H3 (Abcam). A commercially available ECL kit (Advansta, San Jose, CA, USA) was used to reveal the reaction.

### 4.6. Immunoprecipitation

Immunoprecipitation was performed as previously described [2]. Briefly, precleared proteins from cell lysates (250–500 µg) were incubated overnight at 4 °C with anti-Sp1 rabbit polyclonal antibody (D4C3, Cell Signaling, 1:50) or with 4 µg of anti-p53 mouse monoclonal antibody (DO-1, Santa Cruz Biotechnologies). The immunoprecipitated proteins recovered by absorption to EZview Red Protein A Affinity Gel (Sigma-Aldrich) were separated by SDS-PAGE, transferred to PVDF membranes, and probed for the indicated proteins. Three independent experiments were performed.

### 4.7. Notch Agonist DLL4 Binding Assay

Serial dilutions of recombinant human DLL4 (R&D Systems, Minneapolis, MN, USA) ranging between 0.005 µg/mL and 5 µg/mL were prepared in PBS and used to coat multi-well plates by incubation overnight at 4 °C. Negative control wells were incubated with PBS. After removal of solution from each well, 3 × 10^5^ H1975, HCC827 and LC31 parental cells were plated and allowed to bind to coated wells for 72 h at 37 °C in 5% CO_2_. Cells were then washed with ice cold PBS and lysed with RIPA buffer. Whole cell lysates were subjected to SDS-PAGE and western blotting analysis to detect levels of EGFR, Notch1, p53, Sp1, Hes1, and RBPSUH in control and Notch agonist-stimulated cells.

### 4.8. Crosslinking Protein Interaction Analysis

H1975 parental cells were subjected to DLL4 binding assay, as described, and then DMP (dimethyl pimelimidate), a membrane-permeable crosslinking agent (ThermoFisher, Waltham, MA, USA), was added to adherent cells at a concentration of 12 mg/mL. After 1 h incubation at RT with gentle shaking, the reaction was quenched by adding 250 mmol/L glycine for 5 min at RT. Cells were then washed with ice cold PBS, lysed, and subjected to SDS-PAGE and western blotting analysis to reveal the formation of complexes containing p53 and Sp1 in the control and Notch agonist-stimulated H1975 cells.

### 4.9. RNA Interference

Sp1-targeted siRNA pool (ON-TARGETplus SMARTpool siRNA Sp1), TP53-targeted siRNA pool (ON-TARGETplus SMARTpool siRNA TP53), and control non-targeting siRNA pool (CTR) were purchased from Dharmacon Inc. (Lafayette, CO, USA), and used according to the manufacturer’s instructions. Briefly, H1975 and LC31 cell suspension from parental cells were plated at 40% confluence and allowed to grow in a humidified incubator in 5% CO_2_ at 37 °C for 24 h. Cells were then transfected with 100 nmol/L siRNAs using Dharmafect reagent (Dharmacon), as previously described [2]. After 72 h, cells were recovered and then lysed for western blot analysis. Three independent experiments were performed.

### 4.10. Chromatin Immunoprecipitation

ChIP assays were performed using a commercially available SimpleChip Plus Enzymatic Chromatin IP Kit (Cell Signaling Catalog #38191) following the manufacturer’s instructions. Briefly, 1 × 10^6^ H1975 parental cells were counted and allowed to bind to wells pre-coated with 1.5 µg/mL DLL4 or PBS for 72 h at 37 °C in 5% CO_2_. Then unstimulated and DLL4-stimulated cells were fixed in 3.7% formaldehyde for 15 min to crosslink proteins to DNA, and the reaction was quenched by adding glycine for 5 min at RT. Cells were then washed with ice cold PBS and nuclei were isolated and incubated with Micrococcal Nuclease for 20 min at 37 °C to obtain fragmented chromatin. Nuclear pellets were lysed on ice in lysis buffer with protease inhibitors and sonicated with several pulses to break nuclear membranes. Lysates were then clarified by centrifugation at 9400× *g* for 10 min at 4 °C. Then fragmented cross-linked chromatin (5–10 µg) was incubated with anti-Sp1 rabbit polyclonal antibody (D4C3, Cell Signaling) overnight at 4 °C. Protein G magnetic beads were added and incubation was continued for an additional 2 h at 4 °C. Beads were then separated by using a magnetic rack and subjected to serial washes with cold PBS. The immunoprecipitated chromatin was then eluted from beads with gentle vortexing for 30 min at 65 °C, and all samples were incubated with Proteinase K for 2 h at 65 °C. Fragments of DNA obtained from each sample were then purified using spin columns and subjected to real-time qPCR (7900HT Fast Real-Time PCR System; Applied Biosystems) using a SensiFAST SYBR No-ROX kit (Bioline, London, UK). The following primers, recognizing specific regions of the *EGFR* promoter at p53 and Sp1 binding sites, were used [20]: *EGFR* ChIP set 1 (F: 5′-CCCGCGCGAGCTAGACGTCC-3′ and R: 5′-GCTCGCTCCGGCTCTCCC-3′), *EGFR* ChIP set 2 (F: 5′-ACTATGAAGGCTGTTGTCTC-3′ and R: 5′-ACAACAGTGGAACATAAAAT-3′), *EGFR* ChIP set 3 (F: 5′-TCTGTGTTTCTACGGACTGC-3′ and R: 5′-ATGTTTGTGCCTGGGTCT-3′), and *EGFR* ChIP set 4 (F: 5′-AAAGATGTAAGGTTGCTCCC-3′ and R: 5′-TTGGCCAAAAGAAACTGAG-3′). The fold amplification of ChIP samples was calculated using the 2^∆Ct^ (threshold cycle) method, including the normalization to input DNA Ct value following the manufacturer’s instructions.

### 4.11. Statistical Analysis

Statistical analysis was performed using the software MedCalc for Windows, version 10.3.2.0, (MedCalc Software, Mariakerke, Ostend, Belgium) and unpaired Student *t* test was used as appropriate.

## 5. Conclusions

Our findings, taken together, highlighted the role of Notch1 in determining the maintenance or loss of epithelial phenotype in lung cancer cells exposed to EGFR TKIs. Our observations may have translational relevance, since blocking the Notch1 pathway in tumors treated with EGFR inhibitors would prevent downregulation of an oncogene driver, thus maintaining drug sensitivity. Furthermore, since EMT is a reversible process, it would be feasible to recruit back stem-like cancer cells by inducing a reverse driver switch from Notch1 to mutant EGFR, and thus restoring sensitivity to EGFR TKIs.

## Data Availability

Almost all data are included in the manuscript and in the supplementary materials. However, additional data are available from the corresponding author on reasonable request.

## Supplementary Materials

The following are available online at https://www.mdpi.com/article/10.3390/cancers13092022/s1. Figure S1: EGFR mutational status and signaling cascade in parental H1975 cells and H1975-TS by RT-PCR array, Figure S2: Downregulation of EGFR in response to DLL4 binding in HCC827 cells and modulation of Sp1 and p53 transcription factors, Figure S3: Cell toxicity assay in Notch1 overexpressed H1975 cells, Figure S4: Sp1 binding to *EGFR* promoter assessed by ChIP. Figure S5: *EGFR* and *RAS* mutational status in human LC31 cells.
