# Supplementary material for: A Reversible Shift of Driver Dependence from EGFR to Notch1 in Non-Small Cell Lung Cancer as a Cause of Resistance to Tyrosine Kinase Inhibitors
Source: Cancers (Basel). 2021 Apr 22;13(9):2022. doi: 10.3390/cancers13092022 (PMC8122511; doi:10.3390/cancers13092022)
Supplement: Supplementary file 1 [file cancers-13-02022-s001.zip › cancers-1175063-supplementary.pdf]

Supplementary Materials: A Reversible Shift of Driver Dependence from EGFR to Notch1 in Non-Small Cell Lung Cancer as a Cause of Resistance to Tyrosine Kinase Inhibitors

Francesca Iommelli, Viviana De Rosa, Cristina Terlizzi, Rosa Fonti, Rosa Camerlingo, Maria Patrizia Stoppelli, C. Allison Stewart, Lauren Averett Byers, David Piwnica-Worms and Silvana Del Vecchio

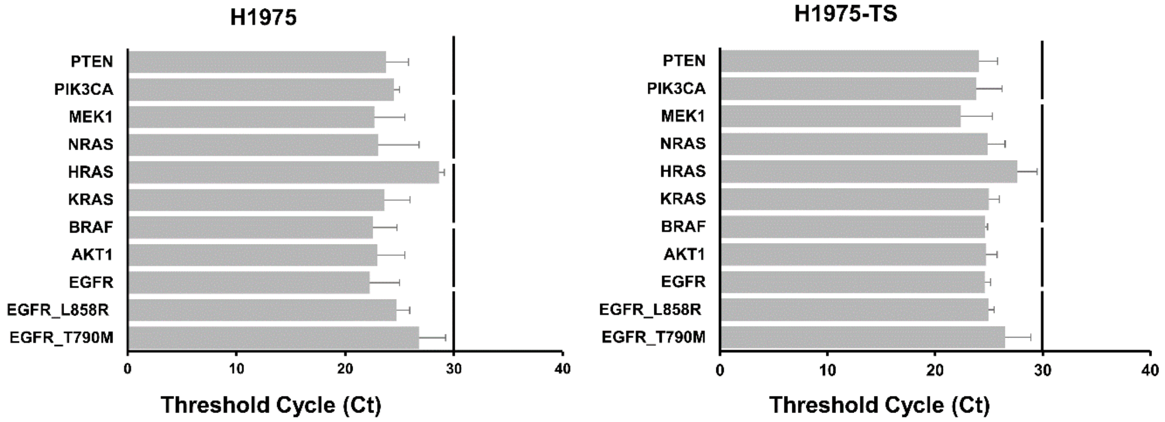

Figure S1. EGFR mutational status and signaling cascade in parental H1975 cells and H1975-TS by RT-PCR array. No significant differences were observed between parental H1975 cells and H1975-TS in threshold cycle (Ct) values of *EGFR*, mutant *EGFR* and 8 additional genes determined by RT- PCR. At least two independent experiments were performed and data are expressed as mean ± SE.

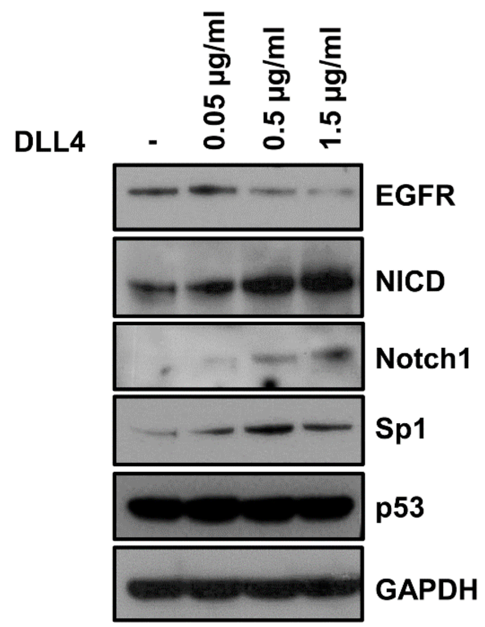

Figure S2. Downregulation of EGFR in response to DLL4 binding in HCC827 cells and modulation of Sp1 and p53 transcription factors. Dose-dependent decrease of EGFR levels in response to DLL4 stimulation for 72 h of HCC827 cells and concomitant upregulation of NICD, Notch1, p53 and Sp1 transcription factors.

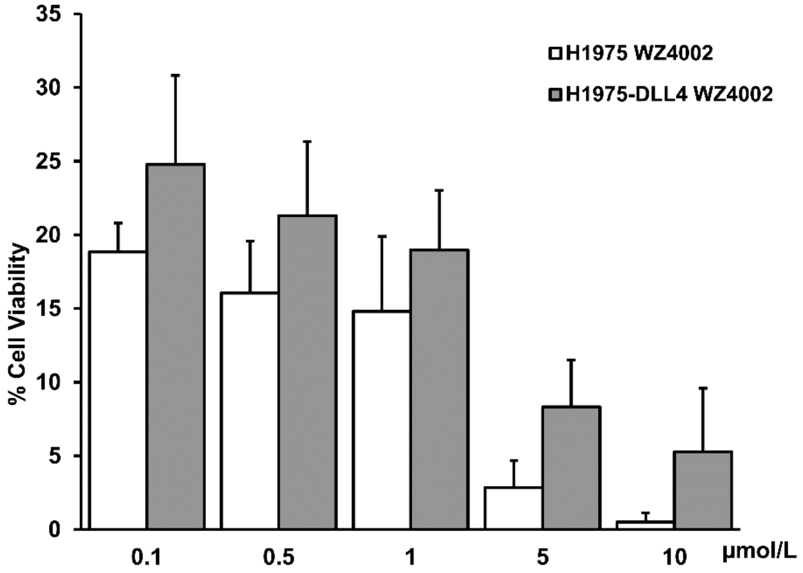

Figure S3. Cell toxicity assay in Notch1 overexpressing H1975 cells. Parental H1975 cells were exposed to DLL4 (1.5 µg/ml) or vehicle for 72 h and then subjected to MTS assay using increasing concentration of WZ4002. Cell viability is expressed as percentage of untreated control cells. At least three independent experiments were performed and data are expressed as mean ± SE.

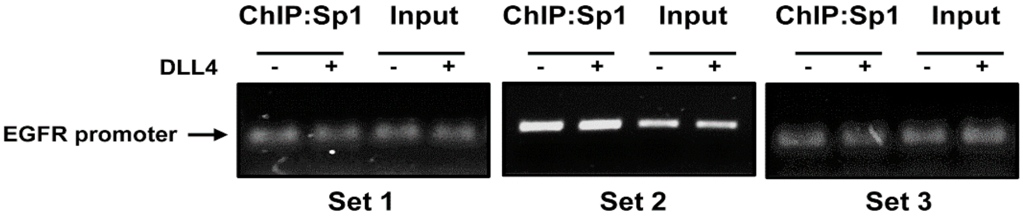

Figure S4. Sp1 binding to *EGFR* promoter assessed by ChIP. ChIP assay was performed by chromatin immunoprecipitation with an anti-Sp1 antibody in unstimulated and DLL4 stimulated H1975 cells. Immunoprecipitated DNA fragments were then amplified using *EGFR* promoter specific primers set 1, 2, 3. At least three independent experiments were performed.

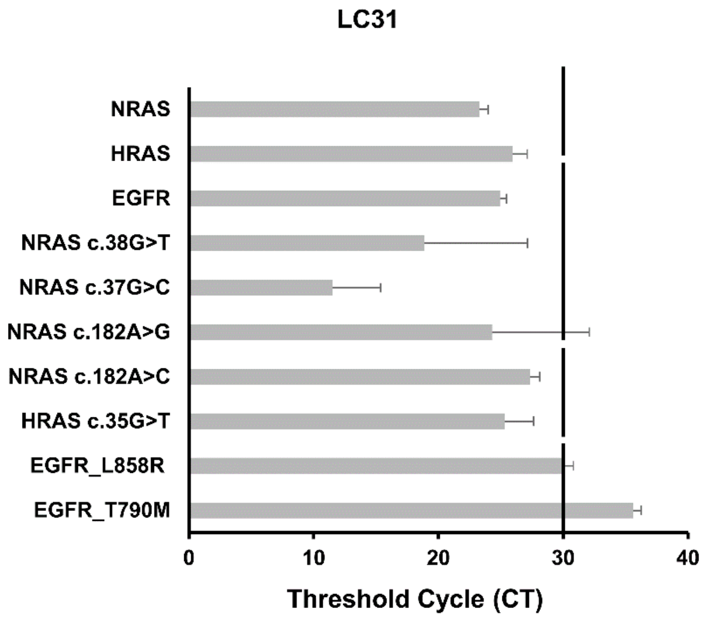

Figure S5. *EGFR* and *RAS* mutational status in human LC31 cells. Threshold cycle (Ct) values of *EGFR*, mutant *EGFR*, *RAS* and mutant *RAS* obtained by RT-PCR array in parental LC31 cells. At least three independent experiments were performed and data are expressed as mean ± SE.
